# Post-Fire Recovery in Coastal Sage Scrub: Seed Rain and Community Trajectory

**DOI:** 10.1371/journal.pone.0162777

**Published:** 2016-09-20

**Authors:** Erin Conlisk, Rebecca Swab, Alejandra Martínez-Berdeja, Matthew P. Daugherty

**Affiliations:** 1 San Diego State University, 5500 Campanile Dr, San Diego, CA 92182, United States of America; 2 The Wilds, 14000 International Dr, Cumberland, OH 43732, United States of America; 3 University of California, Davis, Department of Evolution and Ecology, One Shields Ave, Davis, CA 95616, United States of America; 4 University of California, Riverside, Department of Entomology, 900 University Ave, Riverside, CA 92521, United States of America; US Geological Survey, UNITED STATES

## Abstract

Disturbance is a primary mechanism structuring ecological communities. However, human activity has the potential to alter the frequency and intensity of natural disturbance regimes, with subsequent effects on ecosystem processes. In Southern California, human development has led to increased fire frequency close to urban areas that can form a positive feedback with invasive plant spread. Understanding how abiotic and biotic factors structure post-fire plant communities is a critical component of post-fire management and restoration. In this study we considered a variety of mechanisms affecting post-fire vegetation recovery in Riversidean sage scrub. Comparing recently burned plots to unburned plots, we found that burning significantly reduced species richness and percent cover of exotic vegetation the first two years following a 100-hectare wildfire. Seed rain was higher in burned plots, with more native forb seeds, while unburned plots had more exotic grass seeds. Moreover, there were significant correlations between seed rain composition and plant cover composition the year prior and the year after. Collectively, this case study suggests that fire can alter community composition, but there was not compelling evidence of a vegetation-type conversion. Instead, the changes in the community composition were temporary and convergence in community composition was apparent within two years post-fire.

## Introduction

Disturbance is critical to maintaining community composition and diversity [1]. In Mediterranean-type ecosystems, plant communities have evolved with cool, wet winters and warm, dry summers with periodic fire disturbances during the dry season. A variety of fire-adapted functional types have emerged to allow plants to cope with this disturbance [2]. Recent, primarily anthropogenic, changes to the frequency and severity of fire are impacting the effectiveness of fire adaptations [3], causing declines in abundances of many fire-adapted species, in particular long-lived shrubs [4]. Further, the spread of invasive plants, which can increase with the accumulation of available nitrogen, can also shift the trajectory of post-fire vegetation regeneration [5]. Understanding how fire alters vegetation in fire-prone ecosystems is important given that there are a number of invasive plants, particularly annual grasses, that increase in abundance following fires. In particular, determining which factors, such as microclimate or seed rain, influence species composition following fires is important for helping design effective management strategies.

In Southern California chaparral and coastal sage scrub, fire rotation intervals are 30–40 years [6]. Over the past hundred years, fire return intervals have decreased, especially at the urban-wildland interface where human ignitions are causing more frequent fire [7]. Frequent fire is most likely in areas with mid-level population densities as compared to areas with few individuals per square kilometer or highly urbanized areas with high fire suppression. Coastal sage scrub is an ecosystem characterized by low-growing drought-deciduous shrubs. As a fire-prone ecosystem, it contains many fire-adapted species and is susceptible to changes in fire regimes. In coastal sage communities, more frequent fire has been shown to shift the competitive advantage toward invasive annual plants [8, 9, 10]. Invasive plants, typically annual grasses, begin their growing season earlier and more vigorously than natives [11]. This phenology can allow exotics to pre-empt resources, giving them a “priority advantage” in colonizing recently disturbed sites. Increased nitrogen inputs from deposition of automobile exhaust is likely exacerbating the spread of non-natives in coastal sage habitat [5, 12].

Short-lived annual grasses and forbs grow quickly in the wet season, leaving behind large quantities of dry, combustible biomass for most of the year [13, 14]. This causes higher fuel loads for a larger portion of the year, which can increase fire frequency and spread [15]. This effect has been documented in coastal sage scrub where higher fire frequency has been correlated with higher invasive cover [4, 16]. Thus, increased invasive plant spread can lead to more frequent fire which promotes further invasive plant spread, forming a positive feedback that can result in vegetation type conversion from coastal sage scrub to exotic grass [9, 17]. Once invaded, coastal sage scrub ecosystems require intensive intervention to prevent a new stable state dominated by exotic grasses, exotic forbs, and frequent fire [18].

Understanding the threats to coastal sage scrub communities is critically important as these communities are arguably one of the most endangered ecosystems in the California Floristic Province [19], a biodiversity hotspot [20]. Coastal sage scrub has been reduced to only 10% of its original extent [21], and as a result, is now home to nearly 100 species classified as rare, sensitive, threatened, or endangered [19]. Conversion of coastal sage scrub to exotic grasslands has implications for animal species that depend on coastal sage scrub vegetation. Intact ecosystems with high native diversity promote ecosystem services [22], including resistance to invasion [23], nutrient cycling [24], and food resource availability for wildlife [25]. For example, our study site is home to the federally listed Stephens' kangaroo rat (*Dipodomys stephensi*), which prefers coastal and Riversidean sage scrub habitats with open understories [26, 27]. The Stephens’ kangaroo rat is vulnerable to invasion by exotic grasses, which may reduce their food availability.

To design management actions that prevent type conversion of coastal sage scrub as a result of increased fire frequency, it is important to understand how invaded scrub ecosystems respond to fire. Although a variety of mechanisms guide post-fire plant community regeneration, one rarely quantified aspect of community organization is the contribution of seed rain, where seed abundance plays a large role in determining a species abundance, and seed dispersal is a primary factor affecting seed abundances. Additionally, the physical, chemical, and biological functions of soil are impacted by fire. Post fire soils tend to have greater daily temperature variability, lower humidity, and altered nutrient availability [28]. These factors could affect germination, and thus influence community structure. Therefore, it is important to account for changes in soil and seed rain when evaluating vegetation response to fire. In this study, we attempt to evaluate how plant communities change in a Riversidean coastal sage scrub ecosystem following fire, and what factors influence those changes. In particular, we ask:

1. What are the differences in plant species composition between recently burned and unburned sites?
2. What are the causes of changes in composition?
3. Is community composition changing through time in burned and unburned sites?

To address these questions we compared vegetation composition and seed rain between burned and unburned areas within the same site for two years following a fire event.

## Methods

### Site

The Motte Rimrock Reserve is approximately 300 hectares of predominately Riversidean coastal sage scrub in Southern California’s Riverside County (33.81 N, 117.26 W). It is immediately adjacent to semi-rural residential areas and less than one mile from interstate 215 near Perris, CA. The site sits at roughly 500 m elevation, receives a mean precipitation of 33 cm of rain per year, and has a mean maximum July temperature of 37°C and mean minimum January temperature of 2°C. Typical vegetation of the site includes low-growing aromatic and drought-deciduous shrubs typical of coastal sage ecosystems, such as white sage (*Salvia apiana)*, California buckwheat (*Erigonum fasciculatum)*, and coast brittle-brush (*Encelia californica)*. Over the course of our study, during the 2012 and 2013 growing seasons, the site received approximately half the mean precipitation (17 cm in 2012 and 14 cm in 2013, compared to 42 cm in 2011) and had more moderate temperatures than usual (July max: 33°C in both 2012 and 2013; January min: 9°C in 2012 and 6°C in 2013). The landscape is dominated by granite outcroppings, and has additional nitrogen deposition of around 8–12 kg/ha/yr from car exhaust [29]. Adjacent to the plots, the landscape is highly degraded and invaded, having been the site of a mobile home park in the 1950s. In August of 2011, a 100-hectare fire swept west to east across the reserve. Previous fires at the site last occurred in 1981. No special permits were necessary for access to this University of California Natural Reserve System site and no protected species were involved in the study.

### Plot design and vegetation sampling

**Fig 1:**
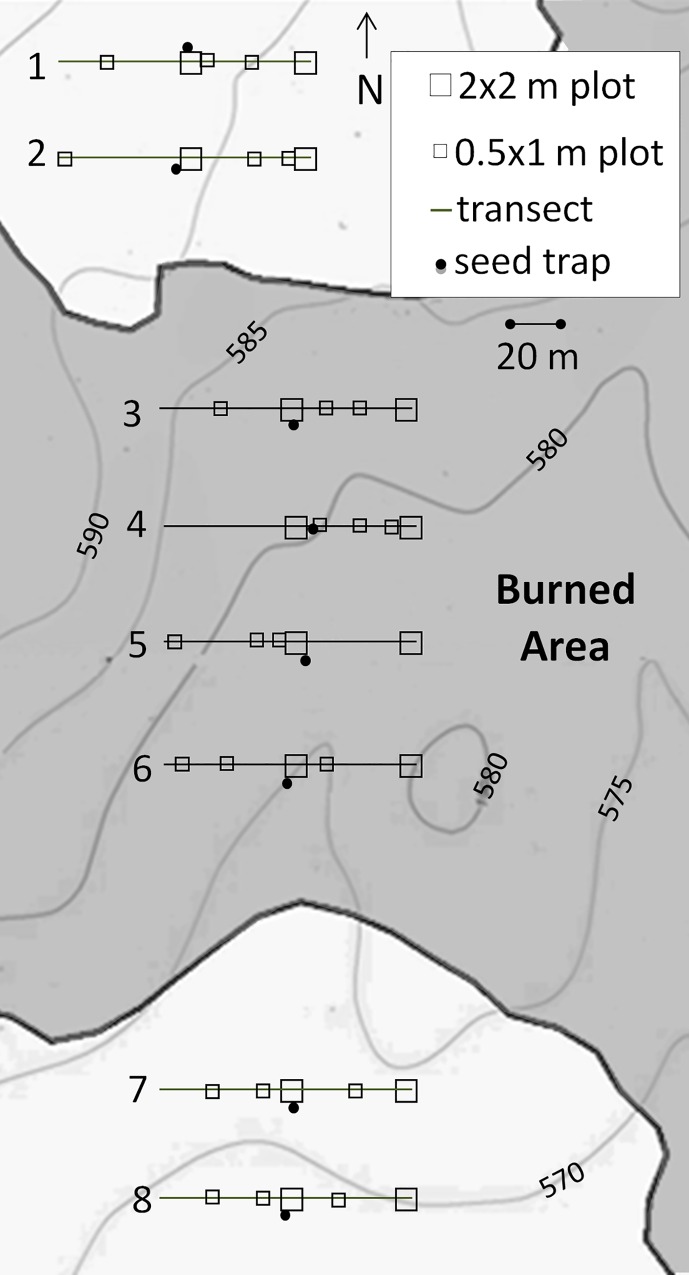
Map of plot and transects on elevation contour map (in meters above sea level). Burned area is shaded.

We established eight west-to-east 100-meter transects: four in the burned area and four in an adjacent unburned area (Fig 1). Transects were parallel to one another and the edge of the burn, and placed 50–100 meters apart. Within each transect, we placed two 2x2-meter plots and three 0.5x1-meter plots per transect. The 2x2-meter plots were placed at the middle and eastern edge of each transect. The 0.5x1-meter plots were placed randomly along each transect. We included two different plot sizes to ensure that species richness didn’t increase dramatically across plot size (as seen in the species area curves for coastal sage in [30]). Plots varied in their slope and aspect, but none were on steep slopes. The plots we refer to as unburned were not burned in this recent fire, however all plots have burned within the last 35 years.

Every four weeks during the growing season (January-May) of 2012 and 2013, we recorded presence and percent cover of each plant species within each plot. For analysis, we defined “cover” to be the maximum cover observed for a particular species across all censuses in a given growing season. Thus, “cover” represents each species at their apex of the season. At one point during the season, we recorded all shrubs intersecting each transect along the entire length. One recorder observed all of the 2x2-meter plots (E.C.) and another recorder observed all the 0.5x1-meter plots (R.S.). Both observers recorded sample plots together prior to measurements to attempt to standardize percent cover measurements.

In 2013, at two locations along each transect we measured temperature at 1 hour intervals with ibuttons (Thermochron DS1921G; Maxim Integrated, San Jose, CA) placed 5 cm below the soil surface. Additional ibuttons (Thermochron DS1923; Maxim Integrated, San Jose, CA), one per transect, measured temperature and humidity above the soil surface.

### Seed rain

In the center of each transect, we placed a seed trap, consisting of a 23 x 28 cm foil tin that contained coarse landscaping gravel 4 cm deep. The gravel kept traps stably in place but allowed seeds to percolate to the bottom of the tin where they were trapped. Every three weeks from the first week in April 2012 until the start of the rainy season in January 2013, we collected all debris that landed in the seed trap. Debris was sorted in the lab using a variety of sieve sizes and a microscope to sort seeds from rocks, soil, and other non-seed material. Seeds were identified to the lowest taxonomic group possible; in some cases seeds could be sorted to species (*e*.*g*., *Erodium cicutarium*, *Emenanthe penduliflora*), but in other cases, seeds were sorted to family (*e*.*g*., *Plagiobothrys*, *Amsinckia*, and *Cryptantha* seeds were pooled under family Boraginaceae) primarily because seed damage made further identification difficult.

### Data analysis

Statistical analyses were performed in R [31]. We compared monthly mean minimum and maximum temperature and humidity in burned versus unburned plots with a linear mixed-effects model (nlme::lme, [32]) with burned treatment and month defined as fixed effects, a random effect of plot identity, and an autoregressive-moving average model to account for temporal autocorrelation. This model structure was needed to account appropriately for temporal autocorrelation stemming from repeated measurements made on the same plots.

To test the difference in overall species richness, we used a generalized linear mixed-effects model (lme4::glmer, [33]) with burned treatment, year, an interaction between burn and year, and plot size defined as fixed effects, a random effect of plot identity, and Poisson error. An early version of the model also included a random effect of transect, but this term was dropped during model simplification because it did not appreciably improve model explanatory power. We tested the possibility of spatial autocorrelation by adding a gaussian correlation structure where each plot was given an x-, y-coordinate describing plots’ spatial distance from one another. Because the addition of the a spatial correlation structure did not change results, and because tests of autocorrelation using Moran’s I suggested minimal autocorrelation unrelated to burn treatment (ape::moran.i, [34]), we report the results in the absence of a spatial autocorrelation structure. Hypothesis testing was performed with a Wald’s *χ*^2^ (car::Anova, [35]).

Next we compared total richness of five functional groups (native shrubs, native annual forbs, native perennial forbs, exotic annual grasses, and exotic annual forbs) between burned and unburned areas across different years. We performed a permutation MANOVA (vegan::adonis, [36]) on a Bray-Curtis dissimilarity matrix. The test partitions multivariate variation according to individual factors in any fully balanced multi-way ANOVA design, with tests done by permutations [37]. Permutations were restricted across plot–the two years of observations were always assigned to the appropriate plot–to account for repeated measures of a given plot. Post hoc tests were performed with Tukey Honestly Significant Difference tests of richness among treatment-year combinations, for each of the functional groups separately (agricolae::HSD.test, [38]).

Similar models were constructed to compare total percent cover, across all species, in burned versus unburned plots using generalized linear mixed-effects model. Non-metric multidimesional (NMDS) scaling (vegan::metaMDS) was then used to visualize changes in vegetation between burned and unburned plots. Percent cover for each of the functional groups was tested with permutation MANOVAs, and we also used a permutation MANOVA to compare seed rain composition between burned and unburned plots. Post hoc tests were performed with Tukey Honestly Significant Difference tests of cover among treatment-year combinations, for each functional group separately (agricolae::HSD.test).

Finally, we investigated changes in community trajectory between years, by comparing seed rain to aboveground plant cover in the year before (first year of the study) and after (second year) seed collection. This was done by first calculating dissimilarity matrices among plots for all taxa (species where possible, and family where seeds could not be identified to species), using the Bray-Curtis index (vegan::vegdist). This analysis was performed for percent cover in 2012, proportion of total seed rain in 2012, and percent cover in 2013. Pairs of dissimilarity matrices were then compared using mantel test (ade4::mantel, [39]), with correlations made between (a) percent cover in 2012 and proportion of total seed rain in 2012, and (b) proportion of total seed rain in 2012 and percent cover in 2013. In such tests, significance is indicative of similarity in taxa relative abundance between the cover and seed rain metrics (i.e. community structure). NMDS was used to visualize the similarity of seed rain arriving at burned and unburned plots in comparison to the vegetation the year before the seed rain (2012) and the year after (2013).

## Results

Environmental conditions differed between burned versus unburned plots. There was significantly higher minimum and maximum mean monthly humidity measured in the burned versus unburned areas (*t* = 3.22, *df* = 6, *p* = 0.018 and *t* = 2.72, *df* = 6, *p* = 0.035 for mean minimum humidity ± standard deviation burned: 23.7±4.7%, unburned: 19.8±8.1% and maximum humidity burned: 85.0±7.1%, unburned: 77.6±8.5%, respectively). However, there was no significant difference in monthly mean daily maximum (*t* = -0.89, *df* = 21, *p* = 0.385) and mean minimum temperature (*t* = -0.28, *df* = 21, *p* = 0.632) between burned and unburned plots.

**Fig 2:**
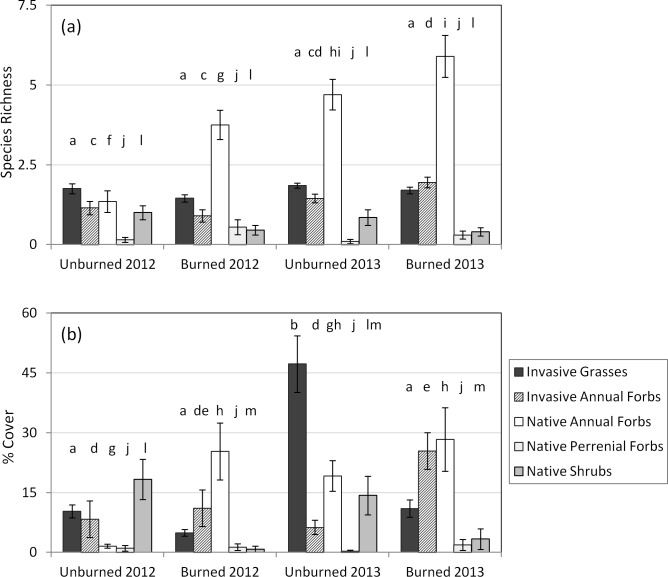
Species richness (a) and cover (b) across all functional types in burned versus unburned plots and in 2012 and 2013. Bars with different consecutive letters denote significant differences (*p* < 0.05) in richness or cover for a given functional group among treatment-year combinations. To clarify that observations within a functional group were compared, a letter is missing between functional groups. Error bars are standard errors.

Overall plant species richness differed significantly between burned and unburned plots (*χ*^2^ = 5.26, *df* = 1, *p* = 0.022), between the two years (*χ*^2^ = 27.80, *df* = 1, *p* < 0.0001), and between plot sizes (*χ*^2^ = 33.40, *df* = 1, *p* < 0.0001). The interaction between burn and year was not significant (*χ*^2^ = 0.71, *df* = 1, *p* = 0.40). Mean total species richness was 20% higher in burned relative to unburned plots (mean ± standard deviation: 8.68 ± 3.82 vs. 7.18 ± 3.39, respectively) and was more than 50% higher in 2013 relative to 2012 (9.60 ± 3.22 vs. 6.25 ± 3.33 respectively). The analysis of relative richness among the five functional types (i.e. native grasses, native forbs, native shrubs, exotic grasses, and exotic forbs), showed significant effects of burning (*F* = 7.70, *df* = 1,75, *p* < 0.001), year (*F* = 14.27, *df* = 1,75, *p* < 0.001), plot size (*F* = 10.49, *df* = 1,75, *p* < 0.001), and an interaction between burn and year (*F* = 4.30, *df* = 1,75, *p* = 0.005). Native annual forbs had the highest richness in the burned plots and showed the greatest difference in richness between years. Exotic forbs richness was also higher in 2013 than 2012 –especially in burned plots (Fig 2A). Native shrub richness was higher in unburned plots, and exotic grass and native perennial richness were relatively more consistent across burning and year combinations (Fig 2A).

Percent cover peaked in early to mid-March for both years in burned and unburned plots. Overall percent cover increased significantly (*χ*^2^ = 39.7, *df* = 1, *p* < 0.0001) from 2012 to 2013 (from mean ± standard deviation: 43 ± 40% to 70 ± 46% in burned plots and from 39 ± 27% to 87 ± 38% in unburned plots). However, there was no significant difference in cover between different plot sizes (*χ*^2^ = 1.61, *df* = 1, *p* = 0.204) or burned versus unburned plots (*χ*^2^ = 0.40, *df* = 1, *p* = 0.525). There was a marginally significant interaction between burn and year (*χ*^2^ = 3.23, *df* = 1, *p* = 0.072). Percent cover of functional groups differed significantly across burn treatment (*F* = 11.94, *df* = 1,75, *p* < 0.0001) and year (*F* = 6.87, *df* = 1,75, *p* < 0.0001), with a significant interaction between burn and year (*F* = 4.00, *df* = 1,75, *p* < 0.0001). Burned plots had more forb cover, especially native forb cover, and unburned plots had more native shrub cover and more invasive grass cover–especially in 2013 (Table 1, Fig 2B).

**Table 1:** Average percent cover of each species in burned and unburned plots.

Species	Code	Unburned	Burned
*Bromus madritensis*	BRMA	25.911	6.597
*Eriogonum fasciculatum*	ERFA	10.666	0.000
*Sisymbrium irio*	SIIR	4.965	10.520
*Plagiobothrys collinus*	PLCO	2.972	0.888
*Schismus barbatus*	SCBA	2.810	1.220
*Salvia columbariae*	SACO	2.620	0.411
*Encelia farinosa*	ENFA	2.348	0.002
*Erodium cicutarium*	ERCI	2.308	7.695
*Stephanomeria exigua*	STEX	1.603	0.164
*Amsinckia menziesii*	AMME	1.523	3.838
*Lessingia filaginifolia*	LEFI	1.145	1.997
*Acmispon glaber*	ACGL	1.117	0.050
*Cryptantha intermedia*	CRIN	0.622	1.941
*Gutierrezia californica*	GUCA	0.563	0.000
*Salvia mellifera*	SAME	0.438	0.014
*Lasthenia gracilis*	LAGR	0.426	3.098
*Mirabilis laevis*	MILA	0.350	0.023
*Solanum xanti*	SOXA	0.331	0.727
*Crassula crenata*	CRAS	0.196	0.181
*Pectocarya linearis*	PELI	0.123	0.088
*Emmenanthe penduliflora*	EMPE	0.113	9.102
*Acmispon strigosus*	ACST	0.058	0.027
*Phacelia distans*	PHDI	0.053	2.058
*Avena barbata*	AVBA	0.037	0.125
*Eucrypta chrysanthemifolia*	EUCH	0.030	2.142
*Croton setigerus*	CRSE	0.005	0.000
*Nicotiana quadrivalos*	NIQU	0.003	0.600
*Tropidocarpum gracile*	TROP	0.003	0.003
*Marah macrocarpus*	MAMA	0.002	0.745
*Phacelia minor*	PHMI	0.000	1.972
*Lupinus bicolor*	LUBI	0.000	0.157
*Malacothamnus fasciculatus*	MAFA	0.000	0.114
*Phacelia ramosissima*	PHRA	0.000	0.059
*Dichelostemma capitatum*	DICA	0.000	0.057
*Erodium botyrs*	ERBO	0.000	0.053
*Oncosiphon piluliferum*	ONPI	0.000	0.002

**Fig 3:**
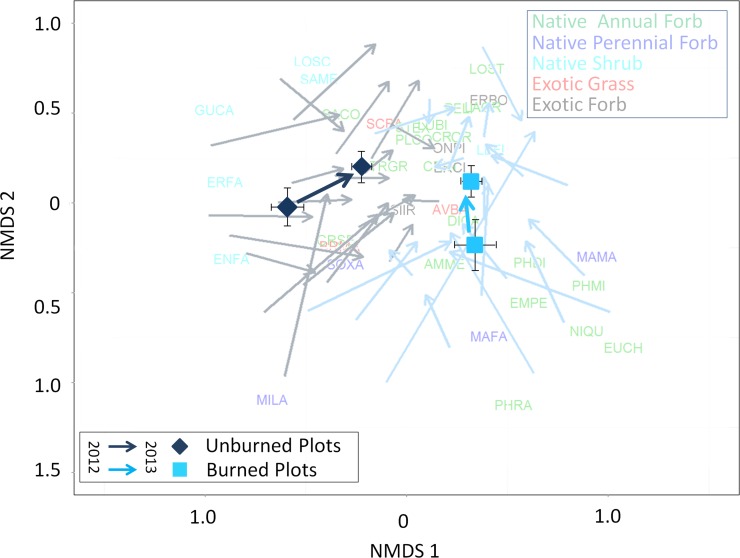
Non-metric multidimensional scaling (NMDS) of vegetation composition (in % aboveground cover for each species) in unburned (dark blue arrows) and burned (light blue arrows) communities, moving from 2012 (tail of arrow) to 2013 (point of arrow) for all plots (faint arrows) and the mean and standard error of the burn by year combination (stress = 0.24). Error bars are standard deviations across plots of the NMDS1 and NMDS2 values. Species abbreviations are defined in Table 1 and color coded by their functional type (green are the native annual forbs, dark blue are native perennial forbs, light blue codes are native shrubs, red codes are non-native grasses, and black codes are non-native forbs). The location of the abbreviation on the plot shows its correlation with the axes.

Dissimilarity in species cover between the two burn treatments and across years can be seen in a non-metric multi-dimensional scaling plot (Fig 3). Burned and unburned sites differed primarily in the degree to which they contain native shrubs versus native forbs. The effect of burning was primarily captured in the first NMDS axis where the species that vary most between burned and unburned plots can be seen on either the far right or the far left of that axis. Burned sites contained considerably more native annuals (*Emmenanthe penduliflora*, *Amsinckia menziesii*, and *Lasthenia gracilis*) and perennial forbs (and native sub-shrub *Lessignia filaginifolia*) while unburned sites contained considerably more native shrubs, in particular *Eriogonum fasciculatum*. Between 2012 to 2013, the unburned plots accumulated more native forbs, and thus most plots within the unburned treatment showed a similar trajectory to each other (Fig 3). The burned plots shared a similar trajectory to each other due to most plots increasing primarily in exotic annuals and shrubs.

**Fig 4:**
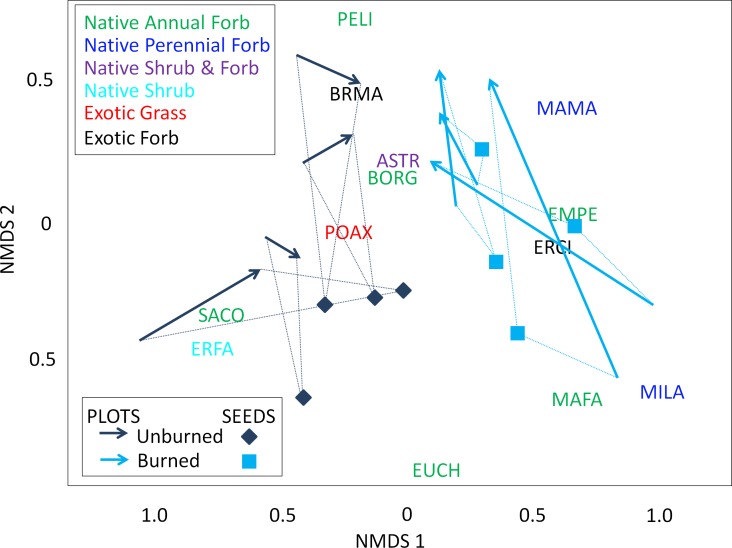
Non-metric multidimensional scaling (NMDS) of unburned (dark blue arrows) and burned (light blue arrows) vegetation communities (in percent cover) in the 2x2 m plots with seed traps, moving from 2012 (tail of arrow) to 2013 (point of arrow). (Stress = 0.16.) The taxa abbreviations (ASTR–Asteraceae, BRMA–*Bromus madritensis*, BORG—Boraginaceae, EMPE–*Emmenanthe penduliflora*, ERCI–*Erodium cicutarium*, ERFA–*Eriogonum fasciculatum*, EUCH–*Eucrypta chrysanthemifolia*, MAFA–*Malacothamnus fasciculatus*, MAMA–*Marah macrocarpus*, MILA–*Mirabilis laevis*, PELI–*Pectocarya linearis*, POAX–*Poeaceae sp*., SACO–*Salvia columbariae*) are color-coded by their functional type (green are the native annual forbs, dark blue are the native perennial forbs, purple are for native shrub and forbs, light blue codes are the native shrubs, red codes are the non-native grasses, and black codes are the non-native forbs).

The composition of seed rain was also different for burned versus unburned plots (*F* = 14.28, *df* = 1,7, *p* < 0.001), with more native forbs seeds in burned plots and more exotic grass seeds in unburned. Furthermore, a strong relationship exists between seed rain and plant cover, irrespective of burning. There were significant correlations between both plant cover in 2012 and seed rain in 2012 (*r* = 0.5251, *p* = 0.019) and seed rain in 2012 and plant cover in 2013 (*r* = 0.6679, *p* = 0.012). In other words, taxa relative abundance in seed rain was predicted by cover the preceding season and predicted cover the next season (Fig 4). The most common seeds occurring in burned and unburned plots were similar to the most common species occurring above-ground within plots. For both burned and unburned plots, exotic grass seeds (not identified to species) were common. Additional common seeds in burned plots were *Erodium cicutarium* and *Emmenanthe penduliflora*. In unburned plots, *Eriogonum fasciculatum* seeds were most common.

## Discussion

Vegetation composition within a Riversidean sage scrub reserve experienced changes in the first two years after a burn: native forb richness and density increased in burned plots and in 2013. The degree to which native forb richness and density increased in burned plots was notable. Numerous studies [8, 9, 10] have found that frequent burning increases density of exotic grasses. Thus, we expected to see more invasive cover in burned plots. Instead, prevalence of exotics decreased following fire.

One explanation for the observed decreased in prevalence of exotics could be the interval between fires was long enough to prevent a vegetation type conversion. The last fire at the site was over 30 years ago. In comparison, resurveys of coastal sage plots originally sampled in the 1930s found that more than half of the sites with type conversions had fires more than once every 30 years [8]. As compared to other Southern California wildlands, our site is surrounded by residential areas with high population density; fires are typically less frequent near centers of high population density due to fire suppression [7].

Another potential explanation for why we did not see an increase in exotic annuals could be the combination of a burn followed by two unusually dry years. In 2012 and 2013, our study site received roughly half the historical mean annual rainfall and followed a year (2011) with above-average precipitation (42 cm). Further, in 2013 approximately 50% of the growing season’s precipitation occurred before December, whereas typically this period accounts for 25% of the annual precipitation. This led to much earlier plant emergence in 2013 as compared to 2012 (although peak biomass occurred in mid-March in both years). Early season rains can cause premature emergence of exotic annuals, resulting in their inability to survive the season [11].

Understanding how fire impacts coastal sage communities is important given the potential for climate change to alter fire intervals. Some models show increased fire frequency in Southern California under PCM and GFDL climate models and A2 and B1 emission scenarios [40]. In the presence of enough rain, exotic annuals could leave more combustible fuel for a fire, potentially increasing fire frequency. Additionally, plant functional traits associated with higher invasiveness are also associated with warmer climates, potentially giving non-natives a competitive advantage with climate change [41]. Thus, invasive grasses and increased fire frequency are interacting threats projected to have an increasing impact on coastal sage scrub ecosystems.

Immediately following a fire, burned and unburned plots varied in species composition, but between 2012 and 2013 species composition in burned plots began to converge on composition in the unburned plots. Further, there is clearly less variability in species composition between plots in 2013 as compared to 2012 (smaller error bars on Fig 3). One possible explanation for the directed and restricted trajectory in plot vegetation composition is that the dry year in 2013, which followed the dry year in 2012, constrained which species were able to survive.

Overall seed abundance was much higher in the burned plots than unburned, as expected. Post fire environments tend to encourage growth and proliferation of seed production as species attempt to grab available niche space and fire followers “refill” the seed bank. *E*. *penduliflora*, a known fire follower, and *E*. *cicutarium* seeds were by far the most common in burned plots. Although *E*. *penduliflora* had extremely high seed numbers in 2012, it was not abundant in 2013. The species is typically only present in the first year following fire [42]. Differences in seed production in burned and unburned plots should converge over time as the recently burned vegetation matures. Additionally, the burned landscape likely promotes higher dispersal distances because of the removal of the vegetation. However, in post fire environments seed predation tends to be higher [43]. These two factors may cancel each other out.

The composition of seeds in the burned plots were quite different from those in the unburned plots. In the burned plots, seed rain seems to be driving the community composition, which would be expected given that the burn removed competition. For three of four burned plots, the seed composition lies between the 2012 and 2013 vegetation composition on the vertical NMDS axis (Fig 4). For example, the seed composition in burned plots is richer in *P*. *linearis*, *M*. *macrocarpus*, Asteraceae and Brassicaceae seeds than was the above-ground vegetation in burned plots in 2012. The result of such seed rain is that the plot-by-plot plant community in 2013 is richer in these taxa. This has important management implications in that it implies broadcast seeding with natives after a fire could shift community composition. We would expect that seed rain would have a big influence on above-ground vegetation in a community recently disturbed, and expect seed rain to have a weaker influence on above-ground vegetation in unburned plots where perennial shrubs dominate and no recent disturbance has occurred.

This study has implications for the endangered Stephens’ kangaroo rat, which occurs in the reserve, and for which current recommendations are for prescribed fires and manual vegetation removal to clear out invasive grasses [27]. Removal of litter is critical to endangered Stephens’ kangaroo rats that require open canopies for foraging [26]. At our site we found that the post-fire vegetation community had decreased exotic grass cover and increased prevalence of bare ground, potentially providing better habitat for Stephens’ kangaroo rats. Additionally, there was higher seed availability in burned plots, which is notable because Stephens’ kangaroo rats need to capture roughly 8 seeds/minute while foraging to meet its high metabolic requirements [44]. Further studies of post-fire vegetation are important for determining the long-term implications of using fire to endangered species management.

This study provides a rarely quantified estimate of the importance of seed rain in structuring the post-fire vegetative community in coastal sage scrub. Our results suggest that there is an impact of burning on above-ground vegetation and seed rain. Seed rain was more important in structuring above-ground communities in burned plots dominated by annual forbs as compared to unburned plots dominated by perennial shrubs. Additionally, back-to-back dry years may have restricted the vegetation composition in burned and unburned plots leading to clear trajectories in vegetation composition and lower inter-plot variability in vegetation composition in 2013. Overall, exotic annuals grasses had lower abundances in burned plots for this study, suggesting that vegetation type conversion is not a certainty, even in highly disturbed plots, if current fire frequency is comparable to historic fire frequency. Implications for management purposes indicate that prescribed burns, done at an appropriate interval, can increase native species and control exotics while increasing habitat for species such as the Stephens’ kangaroo rat. Also, the influence of seed rain on the vegetation community indicates that re-seeding following prescribed burns can impact species presence and abundances.
